# An Ultrafast GPU-Enabled MGVB

**DOI:** 10.3390/proteomes14030034

**Published:** 2026-07-07

**Authors:** Metodi V. Metodiev

**Affiliations:** 1 School of Life Sciences, University of Essex, Colchester CO4 3SQ, UK; mmetod@essex.ac.uk or metodiev1964@gmail.com; 2 Translational Oncology Group, Medical University of Pleven, 5800 Pleven, Bulgaria

**Keywords:** post-translational modifications, proteomics, mass spectrometry, parallel computational algorithms, CUDA C

## Abstract

**Background**: cMGVB is a graphical processing unit (GPU)-enabled implementation of the computational proteomics data analysis toolset MGVB. MGVB was released in 2025 as a Linux program designed to run on multi-node servers. It utilizes a novel algorithm for finding combinations of post-translational modification in peptide MS/MS data. The original combinatorial algorithm required a significant amount of resources to be practical. Hence, the aim of the research reported here was to port the algorithm to GPU and thus increase its speed and efficiency. **Methods**: To accomplish this it was recoded in CUDA C; recursive functions and data structures were re-implemented as non-recursive, and the algorithm was incorporated in a new version of MGVB, now termed cMGVB. **Results**: The re-implemented algorithm is much faster and, unlike the original program, can run on single CPU workstations equipped with inexpensive GPUs and still be much faster than the original algorithm running on HPC clusters. A typical focused search is completed in about a minute by cMGVB compared to 10–15 min by the original implementation. Illustrative case studies are presented and discussed in this report. **Conclusions**: cMGVB enables workflows that were not practical or even possible with the original MGVB.

## 1. Introduction

Over the last 20 years the field of computational proteomics has evolved to encompass diverse experimental approaches, instrumentation and computational algorithms. We are now able to quickly identify and quantify proteins by matching tandem mass spectra to in-silico translated genomic sequences [1,2]. However, one particular area, the comprehensive characterization of post-translationally modified proteoforms, remains problematic. Generally, there are two main approaches to this task: the so called data-dependent, or closed search—exemplified by Mascot [3] and MaxQuant [4]—and the newer open search approaches exemplified by MSFragger [5]. In the data-dependent/closed search, the MS/MS spectra are matched to precompiled peptide precursors; PTMs are included in this compilation and are usually limited to up to 3 different types of modifications for practical reasons. For example, one typically includes N-terminal acetylation; oxidation of Methionine; a common artifact; and phosphorylation on Serine, Threonine, and Tyrosine as variable modifications, i.e., modifications that may or may not affect compatible peptide sites. If different sets of modifications are sought, the search needs to be rerun.

In contrast, the open search strategy does not presuppose a specific set of PTMs but uses a very large precursor mass tolerance, usually as large as 500 Da; the presumed PTM corresponding to the mass difference then needs to be identified post analysis, which usually requires a significant amount of extra effort [5].

Recently a hybrid approach was proposed and implemented in the package MGVB [6,7]. It combines the open precursor search with a combinatorial algorithm, which uses a precomputed delta masses of combinations of PTMs. The combinatorial search is especially useful when implemented in the so called “focused” search, where a fast first search is performed assuming no modifications and missed cleavages to identify the proteins present in the sample. The combinatorial PTM search is then restricted to the proteins identified in the first stringent search. One very good use case for the focused search is the analysis of immunoprecipitated samples or samples obtained by affinity chromatography. In such scenarios the search is focused to proteoforms originating from about a thousand or fewer gene products. The search typically requires less than an hour on an HPC cluster using 24 cores and was at the time considered reasonable.

In more general settings, when all products of the genome need to be considered—often this is when a capture strategy is employed upfront, such as $TiO_{2}$ capture of phosphorylated peptides—the processing time grows in proportion and the analysis becomes impractical unless many more CPUs can be deployed via the message passing parallel mechanism.

To address these limitations, the main aim of this research was to re-implement the combinatorial search as a massively parallel algorithm and offload the heavy computations to the GPU. Such ports to the GPU have been recently shown to significantly accelerate the processing of MS/MS data [8]. Hence, the search algorithm implemented in MGVB was re-coded in CUDA C, and a new program was developed and integrated in the MGVB suite, now named cMGVB. This paper describes the algorithm and presents results from two illustrative case studies. These preliminary studies demonstrate that cMGVB can accomplish the task of identifying PTMs orders of magnitude faster than the original MGVB, which makes previously unfeasible workflows easy to implement.

## 2. Materials and Methods

### 2.1. Algorithms Re-Implementation in CUDA C

This subsection presents a high-level description of the algorithm as implemented in CUDA C and contrasts this implementation with the original CPU-based algorithm, which is detailed in [6]. The source code is not released as of this date, but all object files necessary to build the application are available from GitHub https://github.com/mvm1964/MGVB (accessed on 28 June 2026), and a step-by-step instruction for building is available in Appendix A.

The original algorithm relied on message passing parallelization (by OpenMPI), where subsets of spectra were processed on each CPU independently. For each spectrum in the subset, the local instance of the code performed an open search to select candidate precursors, calculated delta mass for each precursor, matched delta mass to the mod_comb database, assembled modified peptides for the matched PTM, calculated scores and returned the results to the master node for further processing.

cMGVB proceeds in a different way: each individual spectrum is processed by a single thread on the GPU. This way, many thousands of spectra can be processed simultaneously, and a typical focused search is completed in about a minute on a desktop computer equipped with a gaming NVIDIA GPU. To accomplish this the implementation needs to make sure that threads work in lockstep as much as possible. To this end, the following changes were made:

1. Peptide sequences to be searched are encoded as unsigned 8-bit numbers to reduce the memory footprint of the data structures and ensure efficient search on the GPU;
2. All recursive functions used for searching, building fragment sequences and modified fragment sequences, and for calculating scores, are recoded as non-recursive loop-based functions;
3. Candidate fragments for modified sequences are generated by each thread by a non-recursive algorithm, which creates an array of b and y fragments. At this stage only up to three modifications on a peptide sequence are considered;
4. Only singly and doubly charged y and b matches are considered for scoring. Neutral losses will be implemented in future versions of the software;
5. The top five candidates for each spectrum are returned based on raw score.

### 2.2. Workstations Technical Specifications and Software Dependencies

Experiments with cMGVB were done on two platforms: a Ubuntu 22.04.5 LTS workstation with an AMD Ryzen 7 5700X3D 8-Core Processor, with 1 TB disk and 32 GB RAM, and an NVIDIA RTX4070S GPU with 12 GB VRAM, custom assembled by Ozone, Sofia, Bulgaria. In addition, experiments were performed on Codeocean on a Xeon instance with NVIDIA Tesla T4 GPU (https://codeocean.com, accessed on 28 June 2026). Building the program from the provided binary library requires the arbitrary precision libraries GMP (version 6.2.1 was used on the Ubuntu workstation) and MPFR (version 4.1.0 was used on the Ubuntu workstation), and the relational database management system (RDMS) library Sqlite3 (version 3.37.2 was used on the Ubuntu workstation).

The local R version used for post processing was 4.1.2, and the ks package version was 1.15.1.

Experiments with original MGVB were performed on the Ceres cluster at the University of Essex. The two files were analyzed on an 24-cpu instance as previously described [6].

### 2.3. Case Studies

The new GPU-enabled version of MGVB was tested with two datasets, previously described in [6]. One of the data files is generated by LC-MS/MS analysis of immunoprecipitated GFP-tagged Scribble. The MS/MS spectra were generated on an LTQ Orbitrap Velos instrument. A 90-min long gradient was used to separate the digested sample, and a total of 17,310 spectra were acquired and analyzed. This dataset was used to test the performance of cMGVB in high/low mode. The fragment mass tolerance was set to 0.6 Da. The raw data file is available from https://www.proteomexchange.org (accessed on 28 June 2026) with identifier PXD051331.

Another dataset, also described in the first MGVB paper, was used to test the program in high/high mode. This was from another immunoprecipitation experiment where human P53 was isolated together with interacting proteins and analyzed by high-resolution MS/MS on an Orbitrap Eclipse instrument. The dataset contains a total of 99,276 tandem mass spectra. The raw data file is available from https://massive.ucsd.edu (accessed on 28 June 2026) with identifier SV000095580.

Both files were analyzed first by a fast and stringent search to identify the proteins present in the samples. Sequence databases corresponding to the detected proteins were then created and used for a cMGVB search using the same mod_comb table as in the original MGVB publication.

### 2.4. Postprocessing of Peptide Matches and PTMs

Peptide matches were filtered as in the original paper except, instead of using the original C program, an R script was used to calculate kernel densities and PEP (PEP is described in [9]). An SQL script was then used to prepare final table of statistically significant PTMs. Both scripts are provided in the Supplementary Information.

### 2.5. Experiments with MSFragger

MSFragger version 4.4.1 was used in open search mode to analyze the Scribble and TP53 raw data. MSFragger was set to search the same focused fasta files as cMGVB to ensure fair comparison. MSFragger was run on the same Ubuntu workstation. It was set to use 8 parallel threads and 32 GB of RAM. PSMs obtained by MSFragger were filtered at 0.01 FDR using the “expectscore” from MSFragger output files.

### 2.6. Use of Generative AI

The author declares that no generative AI has been used in the preparation of the manuscript and development of cMGVB.

## 3. Results

### 3.1. Implementation, Compilation, and Building

The combinatorial PTM search algorithm described in [6] was successfully re-implemented in CUDA C, and the resulting .c and .cu files were compiled with GCC and NVCC in a collection of objet files and linked with GCC to produce the executable file named mgvb_2026_cuda. All object files and the executable program are available from GitHub at https://github.com/mvm1964/MGVB (accessed on 28 June 2026). To build the executable file on their own machine, one can use object files and execute the command provided in Appendix A.

#### Scribble Immunoprecipitation Analysis

GFP-Scribble immunoprecipitation experiments were described in [6,10]. The raw file used to generate the reported results for Scribble immunoprecipitation is available from https://www.proteomexchange.org (accessed on 28 June 2026) with identifier PXD051331. The results of cMGVB analysis and performance metrics are summarized in Table 1. In terms of identified Scribble peptides, assigned MS/MS scans and identified Scribble PTMs, the results are similar to the ones obtained with the original MGVB algorithm. The slight difference in number of assignments is probably due the fact that cMGVB does not account for neutral losses at the present, which will be amended in future releases. The dramatic difference is in performance metrics. While the original MGVB took more than 10 min on a 24-CPU HPC instance, cMGVB only took 50 s on the Ubuntu workstation equipped with the NVIDIA RTX4070S GPU.

### 3.2. TP53 Immunoprecipitation Analysis

Table 1 also shows a summary of the experiments with a data file obtained in TP53 immunoprecipitation experiments. The raw file used in this research is available from MassIVE https://massive.ucsd.edu (accessed on 28 June 2026) with identifier MSV000095580.

Similarly to the Scribble IP file analysis, cMGVB is more than 17 times faster on the Ubuntu workstation compared to the original MGVB running on 24-CPU instance on the HPC cluster. The speedup is even more dramatic than the speedup of Scribble data analysis, most likely because the TP53 IP data file contains five times more spectra, which can be handled efficiently by the GPU but not so much by the message-passing parallelism employed on the CPUs of the HPC cluster.

### 3.3. Comparisons with MSFragger

The results obtained with MSFragger as a search engine operated in open search mode are also presented in Table 1. MSFragger run times are very similar to cMGVB. It assigns fewer PSMs to Scribble and more PSMs to the TP53 compared to cMGVB, which could be explained by the large difference of spectra in the two datasets. However, MSFragger detected a smaller number of unique peptides for both baits compared to cMGVB.

**Figure 1:**
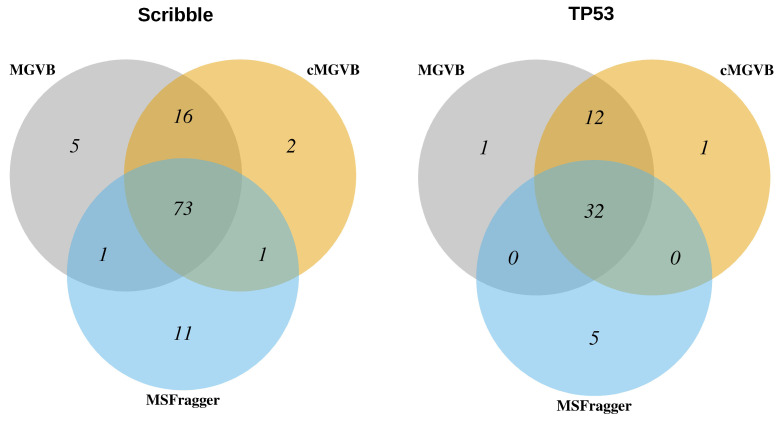
Venn diagrams of Scribble and TP53 unique peptide sequences detected by the three engines. The diagrams were created using the data provided in the Supplementary Information.

Figure 1 presents Venn diagrams of the detected unique peptides for the two bait proteins. The results show very good overlap for cMGVB and MGVB, while MSFragger detects a smaller number of peptides at 0.01 FDR for both Scribble and TP53.

## 4. Discussion

High-resolution mass spectrometry has revolutionized the field of proteomics. It now enables identification and characterization of thousands of proteins—approaching genome-scale analysis—in single experiments. Today’s instrumentation can easily generate more than a million MS/MS spectra in a single day, which sometimes poses a challenge for the downstream analytical pipelines. This challenge is especially pronounced when it comes to mapping post-translational modifications on the identified proteoforms. Unlike DNA and RNA, proteins expressed in eukaryotic cells can carry hundreds of distinct chemical modifications. The importance of some of these modifications in cell biology cannot be overstated. For example, we now know that phosphorylation plays a profound role in the regulation of almost all biological functions, and other modifications are none the less important.

This report introduces a new GPU-enabled implementation of the proteomics toolset MGVB. The emphasis of the changes is on the analysis of post-translationally modified proteoforms by a combinatorial search algorithm, where the new implementation has demonstrated very significant speedup. Although the report describes a working prototype and a fully optimized version will be released later, cMGVB, as the new program is named, is 10–15 times faster than the original MGVB and as fast as MSFragger in focused PTM searches. Within the scope of this report, a focused search is a search on only a subset of gene products, which have been identified to be present in the sample under analysis by a prior stringent search. Such an approach is typical when one is interested in an in-depth characterization of the post-translational modifications on a specific target. In such cases, we would attempt to enrich the target protein, sometimes together with interacting molecules, and then subject the enriched sample to LC-MS/MS analysis. The results section illustrates this with two case studies. In the first we study the polarity regulator Scribble; in the second, the tumor suppressor TP53. In both cases we identify many known and some candidate novel modifications. As previously reported, interacting proteins are also identified in these samples [6,10].

The focused search, however, is not always the best strategy. In many workflows one might be interested in searching against the entire genome of the organism under study. In such cases, the new GPU-enabled cMGVB would provide a decisive advantage over the original MGVB. It makes this workflow feasible. For example, if we were to analyze the two data files under these general conditions, searching against the gene products of the entire human genome, it would take cMGVB about 20 min to process the Scribble data and about 45 min to process the larger TP53 data file. And this is on a desktop computer as described in Materials and Methods. In contrast, the original MGVB would take days to process the files even if as many as 40 CPUs are used.

There are some limitations of the re-implemented program, which need to be highlighted. cMGVB only searches for up to three modified residues in the target peptide. In future releases this will be changed and the number of modifications will be programmable as in the original MGVB. Another limitation is that cMGVB works only on NVIDIA GPUs. In the future, if there is a need, it will be ported to other platforms such as the Apple silicon and AMD GPUs.

Another limitation of the algorithm, as implemented in cMGVB, is that, unlike the original MGVB and Andromeda, it does not consider neutral losses. This will be augmented in future releases.

## 5. Conclusions

General purpose GPU (GPGPU) programming has enabled us to re-implement MGVB to increase the speed of the combinatorial PTM analysis by orders of magnitude. It is now possible to use a single desktop computer equipped with off the shelf gaming GPU and yet complete the analysis many times faster than the original software working on a high-performance computing cluster.

## Figures and Tables

**Table 1:** Performance metrics for cMGVB, MSFragger and MGVB. Execution times, number of spectra assigned to bait protein peptides (modified peptides), unique peptide sequences, and significant PSMs are given for cMGVB run on a Ubuntu workstation equipped with an NVIDIA RTX4070S GPU, MSFragger run on the same workstation, and the original MGVB run on a 24-CPU instance on the Ceres HPC cluster at University of Essex as detailed in Materials and Methods. The results are mean of three replicate runs ± standard deviation. Numbers in parentheses indicate number of spectra assigned to modified peptides.

Performance Metric	Scribble IP	TP53 IP
Number of spectra	17,310	99,276
Search time		
cMGVB	49.7±0.35 s	59.7±0.81 s
MSFragger	53.1±0.25 s	50.3±0.2 s
MGVB	610.3±2.1 s	1070.4±3.2 s
PSMs assigned to bait		
cMGVB	743 (253)	119 (63)
MSFragger	594 (333)	331 (266)
MGVB PSMs	754 (263)	114 (59)
Unique peptides to bait		
cMGVB	92	45
MSFragger	86	37
MGVB	95	45
Significant PSMs		
cMGVB	5749	3226
MSFragger PSMs	3532	3868
MGVB	5701	3303

## Data Availability

All data and software used in the generation of the results reported here are available on GitHub: https://github.com/mvm1964/MGVB (accessed on 28 June 2026). Raw data files are available from https://www.proteomexchange.org (accessed on 28 June 2026) with identifier PXD051331 for Scribble IP, and from https://massive.ucsd.edu (accessed on 28 June 2026) with identifier MSV000095580 for TP53 IP.

## Supplementary Materials

The following supporting information can be downloaded at https://www.mdpi.com/article/10.3390/proteomes14030034/s1, Table S1: cMGVB processing of the Scribble IP data; Table S2: cMGVB processing of the TP53 IP data; Table S3: Scribble unique peptides detected by MGVB; Table S4: TP53 unique peptides detected by MGVB; Table S5: Scribble unique peptides detected by cMGVB; Table S6: TP53 unique peptides detected by cMGVB; Table S7: Scribble unique peptides detected by MSFragger; Table S8: TP53 unique peptides detected by MSFragger; Table S9: MSFragger results for the Scribble IP data; and Table S10: MSFragger results for the TP53 IP data.

## Abbreviations

The following abbreviations are used in this manuscript:

CPU	Central Processing Unit
HPC	Hight Performance Computing
GPU	Graphical Processing Unit
LC	Liquid Chromatography
MS/MS	Tandem mass spectrometry
PSM	Peptide to Spectrum Match
PTM	Post-translational modification

## Appendix A

### Appendix A.1. Building cMGVB from the Provided Binary Library

If the provided executable file is not working on the user’s system, it might be because certain required libraries are either not available or contain incompatible versions. In such cases the executable needs to be built from the provided object files. To build the executable:

1. Make sure GCC, sqlite3, and the arbitrary precision libraries GMP and MPRF are installed on the system.
2. Copy all object files, and the make_cuda.sh file to a new directory.
3. From within this directory execute the following command in terminal:
  ./make_cuda.sh

This will produce the executable mgvb_2026_cuda in the working directory.

### Appendix A.2. Analysing *.mms Files

To analyze the *.mms files available from the codeocean cMGVB capsule, use the main.sh and the sql_script.sql scripts. Edit the scripts before running:

1. Change the file name in main.sh.
2. Change the *.db file name in sql_script.sql.

Consult the comments in the two scripts for more information.

### Appendix A.3. Analysing Raw Files with the New cMGVB Pipeline

To analyze raw files from ThermoFisher instruments, the most convenient way is to use the new focused_cMGVB.sh script, which performs fast first search, creates the necessary database files and then executes the main.sh script as above.
